# An untapped potential for imaging of peripheral osteomyelitis in paediatrics using [^18^F]FDG PET/CT —the inference from a juvenile porcine model

**DOI:** 10.1186/s13550-019-0498-5

**Published:** 2019-03-22

**Authors:** P. Afzelius, O. L. Nielsen, H. C. Schønheyder, A.K.O. Alstrup, S. B. Hansen

**Affiliations:** 1 Department of Diagnostic Imaging, Section of Clinical Physiology and Nuclear Medicine, North Zealand Hospital, Dyrehavevej 29, 3400 Hillerod, Denmark; 2 Department of Nuclear Medicine, Aalborg University Hospital, Aalborg, Denmark; 3 Department of Veterinary and Animal Science, University of Copenhagen, Copenhagen, Denmark; 4 Department of Clinical Microbiology, Aalborg University Hospital, Aalborg, Denmark; 5 Department of Clinical Medicine, Aalborg University, Aalborg, Denmark; 6 Department of Nuclear Medicine and PET, Aarhus University Hospital, Aarhus, Denmark

**Keywords:** Osteomyelitis, [^18^F]FDG PET/CT, Dose reduction, Children, Juvenile pigs, *Staphylococcus aureus*

## Abstract

**Purpose:**

To examine parameters affecting the detection of osteomyelitis (OM) by [^18^F]FDG PET/CT and to reduce tracer activity in a pig model.

**Background:**

[^18^F]FDG PET/CT is recommended for the diagnosis of OM in the axial skeleton of adults. In children, OM has a tendency to become chronic or recurrent, especially in low-income countries. Early diagnosis and initiation of therapy are therefore essential. We have previously demonstrated that [^18^F]FDG PET/CT is promising in juvenile *Staphylococcus aureus* (*S. aureus)* OM of peripheral bones in a pig model, not failing even small lesions. When using imaging in children, radiation exposure should be balanced against fast diagnostics in the individual case.

**Methods:**

Twenty juvenile pigs were inoculated with *S. aureus.* One week after inoculation, the pigs were [^18^F]FDG PET/CT scanned. PET list-mode acquired data of a subgroup were retrospectively processed in order to simulate and examine the image quality obtainable with an injected activity of 132 MBq, 44 MBq, 13.2 MBq, and 4.4 MBq, respectively.

**Results:**

All lesions were detected by [^18^F]FDG PET and CT. Some lesions were very small (0.01 cm^3^), and others were larger (4.18 cm^3^). SUV_max_ was higher when sequesters (*p* = 0.023) and fistulas were formed (*p <* 0.0001). The simulated data demonstrated that it was possible to reduce the activity to 4.4 MBq without compromising image quality in pigs.

**Conclusions:**

[^18^F]FDG PET/CT localized even small OM lesions in peripheral bones. It was possible to reduce the injected activity considerably without compromising image quality, impacting the applicability of PET/CT in peripheral OM in children.

## Background

Osteomyelitis (OM) of long bones may be difficult to treat despite advances in operative techniques and treatment with antibiotics, resulting in considerable morbidity, cost, and sometimes even mortality. The latter is, however, more likely a problem in low-income countries [1, 2]. Cure rates of more than 95% are otherwise achievable with prompt and sufficient treatment [3]. OM has a tendency to become chronic or recurrent. Early diagnosis and initiation of therapy are, therefore, essential to prevent disease progression and to reduce potentially serious complications [4].

The golden standard for diagnosing OM is invasive demanding bone biopsy with histopathologic examination and culturing of the bacteria causing the disease. Imaging on the contrary is non-invasive.

Radiography, computed tomography (CT), and magnetic resonance imaging (MRI) have the advantages of very good spatial resolutions and can show very accurate images of the morphology of anatomical structures. The diagnostic methods using radioisotopes (gamma-camera imaging and positron emission tomography (PET)) have the advantages of showing physiology in vivo, as reflected by the uptake of tracers in the body. Therefore, the techniques are not in competition with each other but complement each other, and by combining modalities like PET and CT, it is possible to acquire both the physiological and the morphological information.

Conventional radiography is unable to diagnose acute OM. OM lesions must extend at least 1 cm and lead to a 30–50% reduction of bone mineral content to generate recognizable radiographic changes [5]. Early findings may be subtle, and changes may not be obvious within the first 5–7 days in children and within 10–14 days in adults. MRI, bone scintigraphy, and CT are central imaging modalities for diagnosing acute OM in children [6], the latter two, however, adding substantially to the radiation exposure of the child. MRI is often considered the best imaging method but is not always available and requires anaesthesia in young children.

Bone scintigraphy demonstrates osteoblastic activity and is considered highly sensitive but not particularly specific [7–9]; however, the reports on the latter diverge, probably depending on the stage of the disease and the age of the patient as well [10]. [^18^F]-fluorodeoxyglucose (FDG) PET may have the highest diagnostic accuracy for confirming or excluding chronic OM in comparison with bone scintigraphy, MRI, and leukocyte scintigraphy. [^18^F]FDG is a glucose analogue containing the radioactive isotope fluorine-18 (^18^F) and is like glucose taken up by inflammatory cells. Most studies have addressed [^18^F]FDG PET for use in the axial skeleton [11, 12], and not in the appendicular skeleton. PET/CT is an invaluable diagnostic tool using ionizing radiation. It is important to be aware of the potential risks of radiation-induced cancer associated with PET/CT examinations and to avoid unwarranted examinations. There is a strong urge to use PET/CT where there is a relevant clinical problem, where the outcome depends on the PET/CT examination, and where non-ionizing alternatives such as MRI and ultrasonic sound examination are inferior.

Children are more sensitive to ionizing radiation than adults [13]. Their cells divide more often, and they live longer after irradiation exposure, allowing potential cancers more time to develop. This consideration has been incorporated into the modern health care system, where all procedures in children involving ionizing radiation exposure are in particular optimized to reduce the dose burden. Most procedures are classical X-ray examinations; however, more advanced examinations like CT and PET are playing an increasing role and are contributing more and more to the overall dose derived from medical procedures. When using imaging modalities in children, radiation exposure should therefore be balanced against the possibility of offering a swift and useful diagnostic procedure, which will depend on the individual case.

We have recently demonstrated that [^18^F]FDG PET was effective in visualizing OM lesions in the peripheral bones of juvenile pigs [14–16]. We also saw that both sequesters and fistulas formed as early as during the first week after inoculation with *Staphylococcus aureus* (*S. aureus*), indicating an unfavourable stage of OM according to the human Cierny-Mader staging [17–19].

OM is most often found in children and elderly [10]. It can be caused by a number of bacteria, but the majority of OM infections are caused by *S. aureus* [4].

In this study, we focus primarily on ionizing radiation originating from ^18^F in a juvenile porcine *S. aureus* OM model, and we simulated different signal-to-noise (SNR) levels in PET images corresponding to various amounts of administered [^18^F]FDG activity. We hypothesize that very low activity levels of injected [^18^F]FDG are sufficient to diagnose OM. If the hypothesis is confirmed, this information is useful for protocol optimization in order to reduce the radiation exposure without compromising the diagnostic value.

## Material and methods

### Pigs and the *S. aureus* model

Twenty clinically healthy, specific pathogen-free Danish Landrace-Yorkshire crossbreed female pigs aged 8–12 weeks were purchased from local commercial pig farmers. After 1 week of acclimatization, the pigs were, under propofol anaesthesia, inoculated with a suspension of a porcine strain of *S. aureus* (S54F9) (10^4^–10^5^ colony forming units (CFU) per kilogram body weight (BW) in 1.0 to 1.5 mL saline) into the femoral artery of the right hind limb simulating haematogenously spread OM in children and avoiding reactive changes induced by the trauma of directly inoculating in a peripheral bone, as described [20].

Most pigs developed clinical signs of infection, e.g., limp in the right hind limb, and after the first pigs, the following were supplied with a single intramuscular (i.m.) injection of procaine benzylpenicillin 10,000 IE/kg (Penovet, Boehringer Ingelheim, Copenhagen, Denmark) to avoid sepsis [21]. Buprenorphine (45 μg/kg Temgesic (Reckitt Benckiser, Berkshire, England)) was given three times daily from the time of inoculation until euthanasia [21]. 1 week after the inoculation, the pigs were anaesthetized and scanned.

After scanning, the pigs were euthanized with pentobarbital (100 mg/kg intravenously) and necropsied, macroscopic lesions noted, and samples collected for histopathology and microbiology. For histopathology, tissues were formalin-fixed for 4 days–30 months, decalcified (bone), dehydrated, embedded with paraffin wax, cut in 3–5 μm thick sections, and stained with haematoxylin-eosin (HE) using standard methods.

The study was approved by the Danish Animal Experimentation Board (no. 2012-15-2934-000123 and no. 2017-15-0201-01239). All facilities were approved by the Danish Occupational Health Surveillance.

### PET/CT scan

The pigs were CT scanned to confirm OM developments 1 day prior to [^18^F]FDG PET/CT scans. [^18^F]FDG was produced by a standard procedure applying a GE Healthcare MX Tracerlab synthesizer, MX cassettes supplied by Rotem Industries (Arava, Israel), and chemical kits supplied by ABX GmbH (Radeberg, Germany). The radiochemical purity was higher than 99%.

Scanning at the Department of Nuclear Medicine and PET Centre in Aarhus was performed with an integrated PET/CT system (Siemens Biograph TruePoint™ 3737 64 PET/CT, Siemens Healthineers, Erlangen, Germany), one bed position spanning 21 cm in the axial direction. The pigs were anaesthetized with propofol and intubated (for mechanical ventilation) and placed in dorsal recumbency as described in [22]. The pigs were placed in the scanners as symmetrical as possible so that the non-inoculated hind limb (left) could be used for comparison. Initially, a scout view was obtained to ensure body coverage of the inoculated limb (right). A CT scan for attenuation correction of PET data was obtained first. PET images were reconstructed using the iterative TrueX algorithm (Siemens), and CT and PET data were co-registered for image fusion by the system. Total activity of 132 MBq [^18^F]FDG was injected in a jugular vein of the two pigs used for simulation, corresponding to 6.2 MBq/kg BW; the other pigs received 6 MBq/kg BW. CT scans for characterization and diagnostics were used, especially in the beginning of the study as the radiation burden was not a limitation and we wanted to make sure that no lesion was overlooked (lesions were primarily identified by osteolysis). Later on, we managed to reduce the mAs to 29 corresponding to an effective dose of approximately 0.2 mSv for scanning the legs without any loss of information on OM. We did not explore this further, as the CT part of PET/CT scanners has improved remarkably with respect to dose reduction since our scanners were installed.

At the Nuclear Medicine Department of Aalborg University Hospital, the pigs were placed in dorsal recumbency position and PET/CT scanned applying an integrated system (GE VCT Discovery True 64 PET/CT 2006, GE Healthcare, USA), one bed position spanning 15 cm. PET images were reconstructed using an iterative algorithm (ViewPoint algorithm (GE Healthcare)) and attenuation correction based on low-dose CT.

### [^18^F]FDG dose simulation

For two pigs (#12 and #13), PET images from Aarhus were retrospectively simulated from the originally acquired data. PET list-mode data of the hind limbs acquired 30–60 min after injection of [^18^F]FDG were histogrammed into 60 time frames each with 0.5 min duration and subsequently reconstructed using the TrueX iterative algorithm. Four sets of PET images with a varying signal-to-noise level were generated by summing 2, 6, 20, and 60 time frames symmetrically around the time point 45 min after tracer injection. Thereby, the four sets of images represented 1, 3, 10, and 30 min effective scan time at an average time point of 45 min post-tracer injection. For a constant scan time of 30 min, these images would represent the image quality obtainable with an injected activity of 132 MBq, 44 MBq, 13.2 MBq, and 4.4 MBq respectively.

### Reading the scans

PET with [^18^F]FDG and CT were read individually. PET was also read as fused images with CT. All scans were evaluated by an experienced specialist in nuclear medicine and CT on a Dell Precision T7400 working station (Dell, Austin, TX, USA) using the Philips Extended Brilliance™ Workspace V.4.52.40140 EBW-NM 2.01 software (Philips Health care, PC Best, The Netherlands).

### Microbiology

The inoculums were prepared from the S54F9 strain of *S. aureus*, isolated from a chronic embolic pulmonary abscess in a pig [23]. The strain is pan-susceptible, and the clinical signs of OM were modifiable with procaine benzylpenicillin. The target inoculum was 10^4^ CFU/kg BW and was inoculated in the femoral artery of the right hind limb 7 days prior to the scans. Swabs and tissue biopsies were obtained postmortem for bacteriological culture to confirm the bacterial agent, although *S. aureus* was identified in a few lesions by immunohistochemistry as described previously [24].

### Statistics

A two-tailed Pearson test was used for correlations, and for comparison of groups, a Mann-Whitney *U* test was used. A *p* level of < 0.05 was considered statistically significant.

## Results

**Fig. 1:**
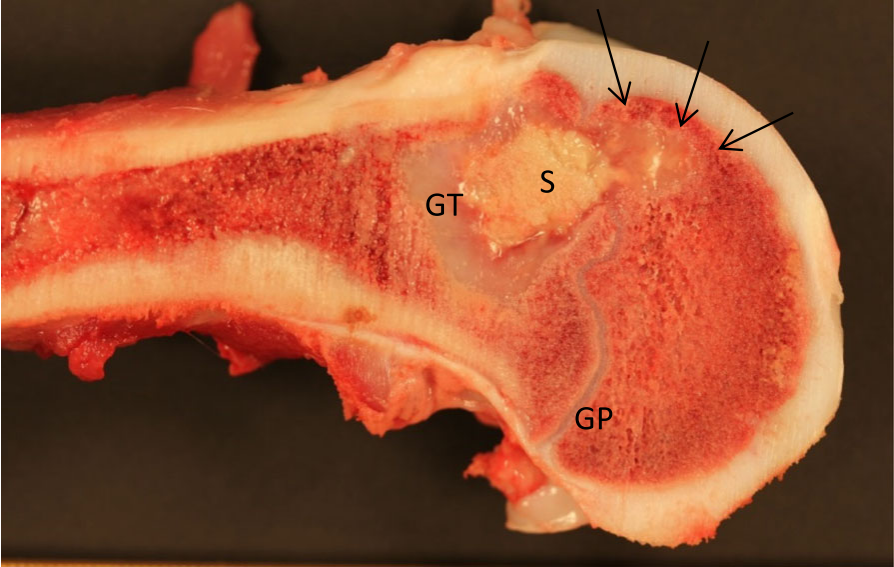
Mid-sagittal cut through the distal femoral bone of the right hind limb of a pig infected by the inoculation of *S. aureus* into the femoral artery. Pathological chronic, purulent, sequestering osteomyelitis (S) is seen in the metaphysis with disruption of the growth plate (GP) and affection of the epiphysis (arrows). The lesion has a size (diameter) of approximately 2 cm. The thick peripheral zone of granulation tissue (GT). Pig 4, 14 days after the inoculation

After the inoculation with *S. aureus*, the pigs developed 56 OM lesions in different anatomical locations in the field covered by CT. The OM lesions were diagnosed by the presence of the main osteolysis by CT. Most lesions were also identified during necropsy revealing concordance between gross pathology changes and the CT diagnoses (Fig. 1). Microbiology (re-isolation of the inoculated *S. aureus* bacterium) was performed at least in one lesion from each of the pigs, although *S. aureus* was identified in a few lesions by immunohistochemistry (Table 1). Tissues were collected for histopathology, and descriptions (subacute OM) have been presented, along with other findings, for some of the pigs in previous papers (see references in Table 1).

**Table 1:** Findings by CT, PET, necropsy, and microbiology of the individual pigs. Fifty-five of the OM lesions were found in the right hind limb, and one was found in right humerus

Pig^a^	Number of OM (CT)	Number of OM (PET)	Number of OM (necropsy and/or histology)	Microbial *S. aureus*	Vol. ranges cm^3^	SUV ranges g/ml	FDG activity MBq/kg
1^a^	4	4	3	Yes	0.16–2.54	2.6–4.4	7.48
2^a^	1	1	1	Yes^c^	2.05	3.9	9.73
3^a^	2	2	1	Yes^c^	0.02–0.23	5.2–5.7	9.36
4^a^	1	1	1	Yes	3.76	2.4	18.86
5^a^	3	3	3	Yes^c^	0.42–2.41	4.0–7.3	18.86
6	5	5	5	Yes	0.07–0.36	2.3–5.9	17.09
7^a^	4	4	4	Yes	0.17–0.73	2.9–4.5	22.09
8^a^	5	5	6^b^	Yes^c^	0.33–0.59	10.5–16.3	22.42
9^a^	2	2	1	Yes^c^	0.01–0.29	3.4–9.0	6.10
10	3	3	1	Yes^c^	0.01–4.18	4.0–9.2	6,67
11	4	4	3	Yes^c^	0.07–0.36	3.0–6.4	6.23
12	3	3	2	Yes^c^	0.21–1.52	2.4–8.0	5.97
13	2	2	1	Yes	0.29–0.79	4.6–5.9	5.0
14	4	4	4	Yes^c^	0.25–0.45	4.4–5.1	3.98
15	3	3	3	Yes	0.09–0.36	3.0–6.1	5.18
16	3	3	3	Yes	0.03–1.37	1.0–3.9	5.55
17	1	1	1	Yes^c^	0.03	6.1	5.42
18	2	2	2	Yes^c^	0.58–2.28	7.8–10.1	5.0
19	3	3	3	Yes^c^	0.05–3.84	4.0–7.8	5.85
20	1	1	2^b^	Yes^c^	0.01	3.6	4.86

^a^Some data from these pigs have been published previously: pigs 1–3 [14, 15], pigs 5–8 [16], and pigs 1–9 [40, 41]

^b^Pigs 8 and 20 were diagnosed with osteomyelitis by necropsy and/or histology disclosing affection of the proximal and intermediary phalanges of toe no. IV in pig 8 and the intermediary and distal phalanges of toe no. V in pig 20; by scanning, only one of the lesions in each of the pigs was identified

^c^*S. aureus* identified by cultivation from a periosseous abscess (pigs 2, 3, 9–12, 14, 17–18), from an arthritic lesion (pig 8), from an inoculation site abscess (pig 19), and by immunohistochemistry (pigs 5 and 20)

**Fig. 2:**
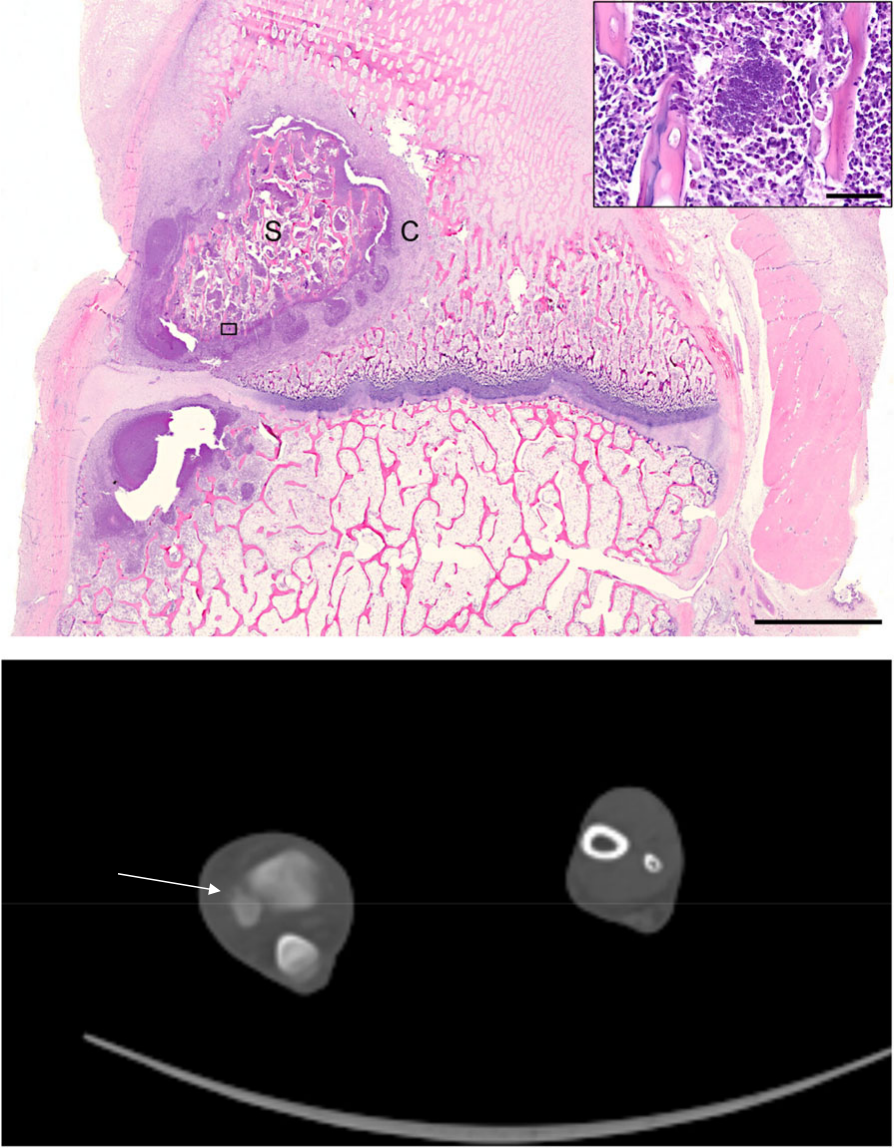
Above: Histopathology, a mid-sagittal section of distal right fibula of pig 11. Pathological subacute osteomyelitis in the cranial aspect of the bone located both distal (abscess) and proximal (suppuration with sequestration) to the growth plate. Sequester (S) and peripheral capsule (C). Bar 2 mm. Inset: bacteria and a necrotic bone trabecula. Bar 40 μm. HE stained. Below: A CT scan of the OM in the distal right fibula

**Fig. 3:**
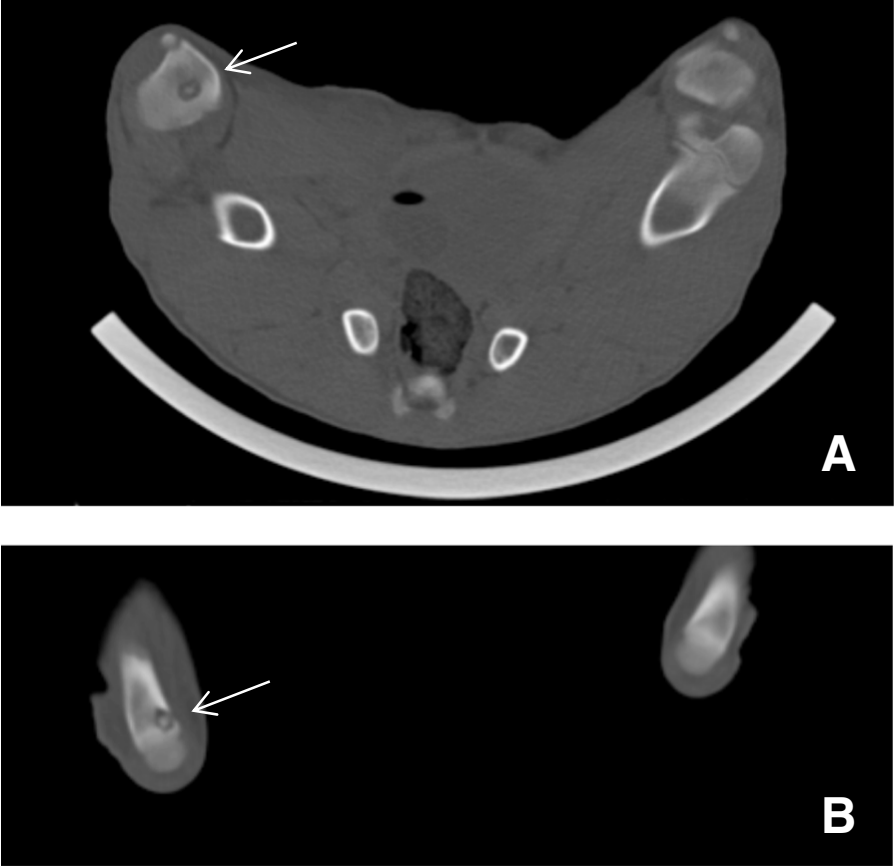
A lesion in pig 6 with sequester formation marked by an arrow in the right proximal tibia (**a**). A lesion in pig 6 with both sequester and fistula formation marked by an arrow in the right calcaneus (**b**)

Of the 56 OM lesions, 1 OM lesion developed in the right humoral bone and 55 OM lesions developed in the right hind limb. The OM lesions most often involved the growth zones of the long bones (Figs. 1 and 2). All lesions were recognized on CT (osteolysis, sequesters, fistulas, and cortical destructions (Figs. 2 and 3)). The lesion size ranged from 0.01 cm^3^ to 4.18 cm^3^, but all accumulated [^18^F]FDG PET (Table 1). The volume of 0.01cm^3^ seems to be about the limit for evaluation on CT, and the FDG-accumulation helped the interpretation. A small lesion of 0.07 cm^3^ seen on CT was examined histopathologically to confirm the existence of OM and the presence of coccoid bacteria (Fig. 2).

By reading the [^18^F]FDG PET/CT, 49 of the 56 OM lesions were identified as having formed sequesters and 44 had formed fistulas (Table 2)*.* Four lesions formed neither sequesters nor fistulas 1 week after the inoculation with *S. aureus* (Table 2)*.* In three cases of fistula formation, there were no signs of sequester formation on CT. The lesions were however very small (0.01 and 0.02 cm^3^).

**Table 2:** Numbers of osteomyelytic lesions among 56 lesions (20 pigs) with fistula and sequester formations judged by CT

OM with sequester	OM with fistula	OM with both sequesters and fistula	OM without neither sequesters nor fistulas
49	44	41	4

Maximal standardized uptake value (SUV_max_) was higher in the group that formed sequester than in the group that did not, 5.2 versus 3.9 g/mL (*p* = 0.023), and SUV_max_ was also higher in the group that formed fistulous tracts than in the group that did not, 5.6 versus 3.3 g/mL (*p* < 0.0001). The volume of the OM lesion was similar whether the OM had formed fistulous tracts or not (*p* = 0.216), whereas sequester formation was more often seen in the larger OM lesions than in the smaller lesions (*p* < 0.0001). (SUV_max_) was higher in OM with fistula formations than in OM with sequester formations, 4.7 g/mL versus 3.4 g/mL (*p* = 0.002).

**Fig. 4:**
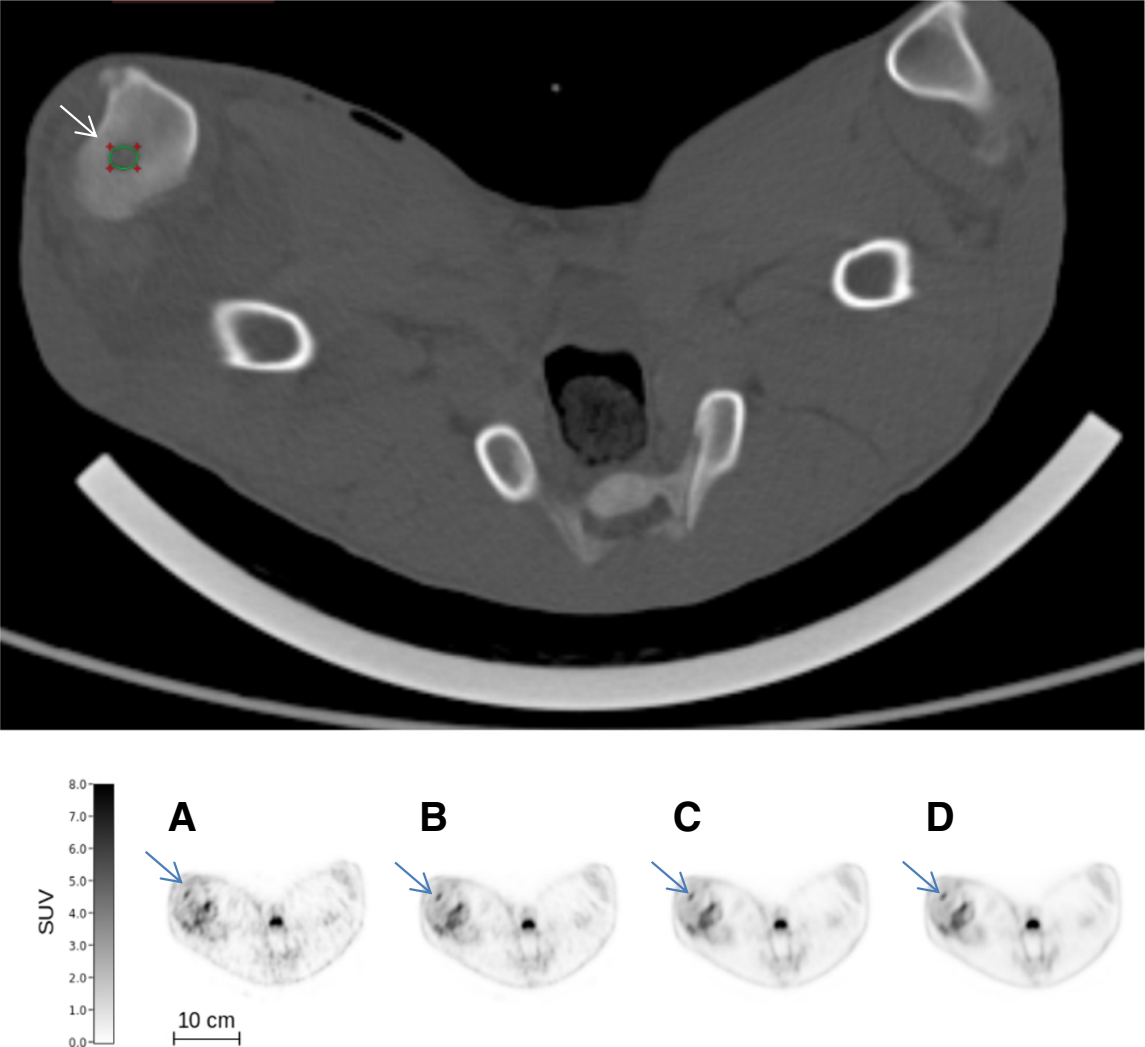
Above: CT scan (bone window) of a small (0.21 cm^3^) osteomyelytic lesion (osteolysis) in right proximal tibia of pig 13 (indicated by an arrow). Original SUV_max_ on Philips EBW was 2.4 g/mL. Below: Increasing activities of [^18^F]FDG in the osteomyelytic lesion of the right proximal tibia (indicated by arrows): 4.4 MBq (**a**), 13.2 MBq (**b**), 44 MBq (**c**), and 132 MBq (**d**) simulated injected activity

**Fig. 5:**
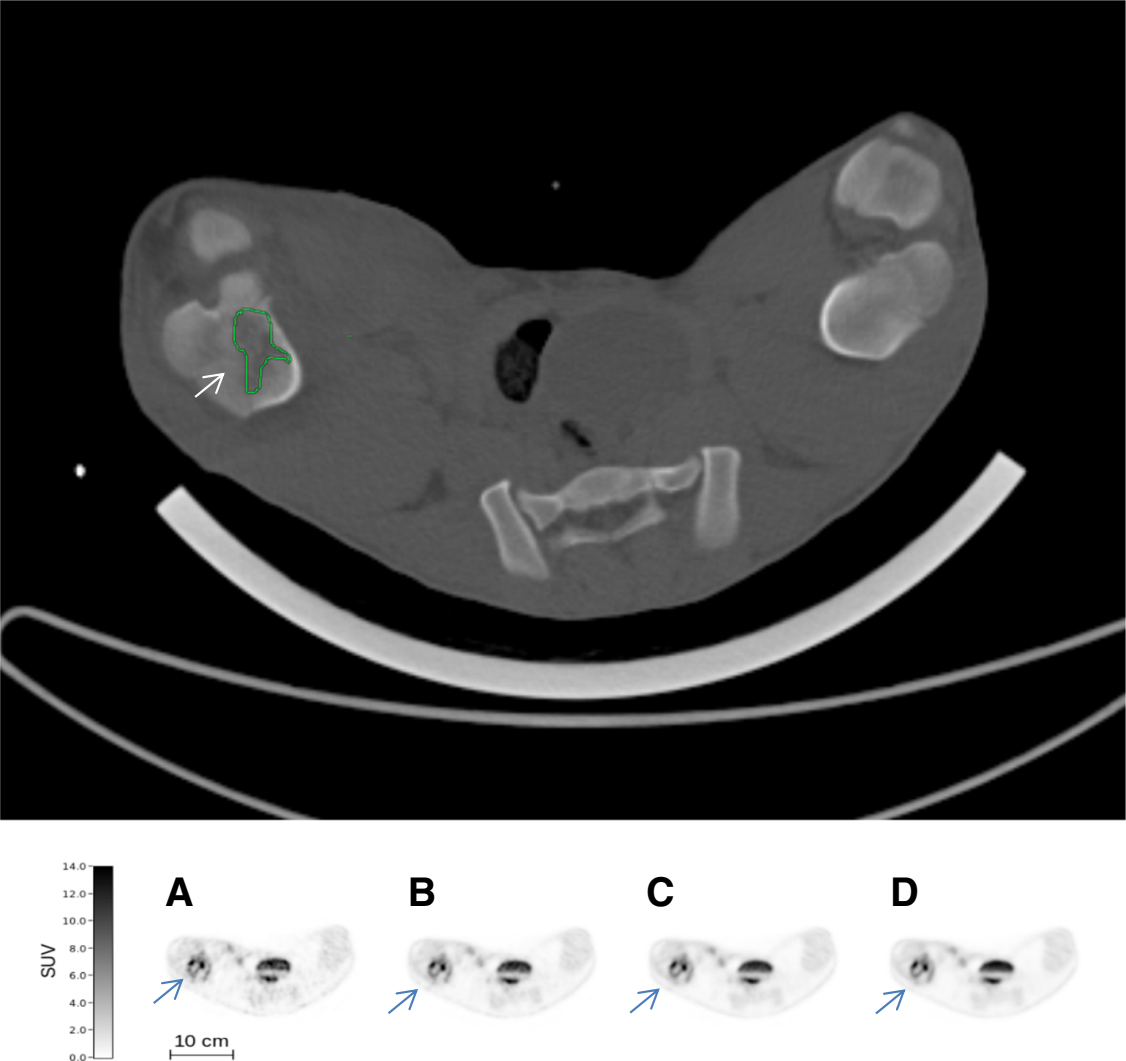
Above: CT scan (bone window) of the larger medial, irregular (1.52 cm^3^) osteomyelytic lesion in the right distal femur of pig 13 (indicated by an arrow). Original SUV_max_ on Philips EBW was 8.0 g/mL. Below: Increasing activities of [^18^F]FDG in the osteomyelytic lesion in the right distal femur (indicated by arrows): 4.4 MBq (**a**), 13.2 MBq (**b**), 44 MBq (**c**), and 132 MBq (**d**) simulated injected activity

The simulated variation of [^18^F]FDG activity (132 MBq, 44 MBq, 13.2 MBq, and 4.4 MBq) demonstrated that for 2 pigs, it was possible to reduce the activity of [^18^F]FDG to 4.4 MBq for both smaller and larger lesions, thereby reducing the dose of ionizing radiation. This corresponded to an injected activity as low as 0.19 MBq per kilogram BW. Examples of both a small and a large OM are shown in Figs. 4 and 5, respectively. The visual differences in the PET images were minimal although the simulation represents a 30-fold variation of the injected activity.

## Discussion

Although considered routine in adults, PET/CT in children has been somewhat limited. As in adults, unnecessary examinations should be avoided. Given a large number of [^18^F]FDG PET, and especially CT, examinations performed annually throughout the world, reduction of the administered activity without compromising the clinical information being sought is encouraged. The decision to utilize PET/CT in children should be made individually, taking cumulative radiation exposure and the benefit of the scan into account. For all nuclear medicine examinations, the optimum administered activity is a trade-off between radiation dose and the image SNR, which is defining the obtainable clinical sensitivity. The recommended maximum injected activity of [^18^F]FDG for paediatric use is in the range of 3.7 to 5.2 MBq/kg (0.10–0.14 mCi/kg), with a minimum injected activity of 26 MBq (0.7 mCi) [25]. We have demonstrated that in OM diagnostics, it is possible to reduce the injected activity of a radiotracer even more as our simulation study indicated that even small lesions are clearly visible at an injected activity as low as 0.19 MBq/kg BW (0.005 mCi/kg)). This supports the findings by Gatidis et al. in a PET/MRI study [26].

Many diagnostic tools are available, but no single test has 100% diagnostic accuracy [27]. It may have been easier for our imaging specialist to recognize even small OM lesions on CT scans since the time for interpretation of CT scans was unlimited, and we only examined juvenile animals that were otherwise healthy prior to inoculation. Children, in general, also suffer less from degenerative disorders in joints and bones than adults do, thus leading to the less competing glucose uptake in surrounding tissues which makes the interpretation of PET easier in children. Thus, we believe that our results in the pig model translate well, supporting the application of CT and reduced dosage of [^18^F]FDG in children to diagnose OM. By using the lower suggested activity of [^18^F]FDG for PET/CT, the radiation exposure in children will be lower than for [^99*m*^Tc]MDP/HDP bone scan, which further speaks for using PET/CT.

Johansen and Jensen have reviewed the haematogenously spread *S. aureus* OM in animal models [20]. This group and others have noticed a similarity to children where OM most often involve the growth zones of the long bones of the lower extremities, perhaps due to more exposure of the long bones of the extremities to small blunt trauma from childhood activities, especially of boys, creating a locus of minor necrosis [28, 29]. Our studies show similar seeding of *S. aureus* in the growth zones of juvenile pigs as reported previously [14, 15]. This may be due to the characteristic blood supply in long bones of juveniles [10]. OM is most often localized to the lower limbs of children, and 50 % of cases occur in those less than 5 years of age, twice as frequent in males as in females [10]. This could support the argument for applying limited CT of the limbs in older children, which would reduce radiation exposure significantly. While OM typically is solitary, multifocal bone involvement occurs in 7% of children and in 22% of neonates [10]. In neonates, there may be a need for whole body examinations. It may be necessary to perform the scans with some degree of anaesthesia in some children, which could reinforce the idea of initiating the hunt for OM with the quicker CT.

The spatial resolution of our Siemens CT is about 1 mm and with PET about 4 mm. Since the concept of SUV is a normalization of the image voxel values to variation in injected activity and body weight, the apparent SUV_max_ is not expected to depend upon the tracer activity. However, obviously, the image noise level will increase when the activity is reduced (Poisson statistics). With the current generation of PET/CT scanners, 0.19 MBq/kg BW may thus be about the limit for the interpretation of FDG accumulation in OM.

Especially, small lesions in CT and PET images are affected by the partial volume effect (PVE) [30–32]. The quantitative accuracy of PET images may thus be questionable as the spatial resolution is low (4–6 mm) compared to CT and MRI (< 1 mm) and the tissue sampling is limited. We have not made corrections for PVE as we compared with the normal anatomical site in the other limb when detecting OM lesions. The size of all OM lesions was measured on CT and thereby not affected by the limited spatial resolution of PET. To minimize PVE, we used SUV_max_ instead of the volumetric mean SUV. In clinics, the detection of an OM lesion is more important than the measurement of quantitative PET-tracer uptake. In recent years, a lot of effort has been put into the development of new CT scanners and scanning protocols that markedly reduce the absorbed dose without compromising the diagnostic performance and the image quality [33–39]. In the pigs, the glucose metabolism was significantly higher in areas of fistula formation than in areas of sequester formation, indicating the attraction of cells with higher glucose metabolism in the former conditions. Probably, fistulation will reduce the intraosseous pressure in the cortical bones easing the perfusion, the inflammatory process, and the glucose uptake of reparative cells.

The elements normally considered to characterize chronic OM such as the presence of a sequestrum and/or a fistulous tract were very frequent and present as early as during the first week of infection in our pig study. Thus, the differentiation between acute and chronic OM in humans may be different in juvenile pigs indicating more aggressive pathogenesis in juvenile pigs, or that the progression of the disease in children may be faster than assumed.

To summarize, we believe that reduced dosage of [^18^F]FDG and limited bone localization CT may be useful in children in selected cases. It would be very interesting to examine these aspects in young children as the limitation of this study, obviously, was the use of an animal model. To accurately determine the optimum administered activity for paediatric OM protocols, however, a dedicated receiver operating characteristics (ROC) study in children will have to be performed.

## Conclusions

In conclusion, [^18^F]FDG/CT helps to localize very small OM lesions in peripheral bones. This supports the use of PET/CT, also in children, as it was possible to reduce the injected activity markedly.

It would be interesting to see if the results obtained in our animal model will be relevant in clinical practice of imaging of the haematogenously spread *S. aureus* OM in peripheral bones, preferably in young children.

## Abbreviations

BW: Body weight
CFU: Colony forming units
CT: Computed tomography
FDG: [^18^F]-fluorodeoxyglucose
HE: Haematoxylin-eosin
i.m.: Intramuscular
MRI: Magnetic resonance imaging
OM: Osteomyelitis
PET: Positron emission tomography
ROC: Receiver operating characteristics
*S. aureus*: *Staphylococcus aureus*
SNR: Signal to noise
SUV_max_: Maximal standardized uptake value
